# Supplementary material for: Heat adaptation of phage T7 under an extended genetic code
Source: Virus Evol. 2021 Dec 1;7(2):veab100. doi: 10.1093/ve/veab100 (PMC8923235; doi:10.1093/ve/veab100)
Supplement: veab100_Supp [file veab100_supp.zip › Sequencing_Analysis.zip!/Sequencing_Analysis/A2/evidence/JC_SIDE_1_47.html]

BRESEQ :: Evidence


| New junction evidence | | | | | | | | | | | |
| --- | --- | --- | --- | --- | --- | --- | --- | --- | --- | --- | --- |
|  | | seq id | position | reads (cov) | reads (cov) | score | skew | freq | annotation | gene | product |
| \* | ? | NC\_001604.1 | = 7781 | 452 (0.990) | 10 (0.020) | 7/558 | 19.9 | 2.3% | intergenic (–/–) | *–/–* | –/– |
|  | ? | NC\_001604.1 | 7899 = | 446 (1.050) |  |  |  |  | intergenic (–/–) | *–/–* | –/– |
| Rejected: Coverage evenness skew score above cutoff. | | | | | | | | | | | |
| Rejected: Frequency below/above cutoff threshold. | | | | | | | | | | | |

Only 100 of 457 total aligned reads displayed.  

`CTGATGGCTCTTTGCGGCACCCATCGTTCGTAATGTTCCGTGGCACCGAGGACAACCCTCAAGAGAAAATGTAAT‑‑CACACTGGCTCACCTTCGGGTGGGCCTTTCTGCGTTTATAAGGAGACACTTTATGTTTAAGAAGGTTGGTAAATTCCTTGCGGCTTTGGCAGCTATCCTGACGCTTGCGTATATTCTTGCGGTATACCCTCAAGTAGCACTAGTAGTAGTTGGCGCTTGTTACTTAGCGGCAGTGTGTGCTTGCGTGTGGAGTATAGTTAACTGGTAATACGACT‑‑CACTAAAGGAGGTACACACCATGATGTACTTAATGCCATTACTCATCGTCATTGTAGGATGCCTTGCGCTCCACTGTAGCGATGATGATATGCCAGATGGTCACGCTTAATACGACTCACTAAAGGAGACACTATATGTTTCGACTTCATTACAACAAAAGCGTTAAGAATTTCACGGTTCGCCGTGCTGACCGTTCAATCGTATGTGCGAGCGAGCGCCGAGCTAAGATACCTCTTATTGGTAACACAGTTCCTTTGGCACCGAGCGTCCACATCATTATCACCCGTGGTGACTTTGAGAAAGCAATAGA  >  NC_001604.1/7481‑8081`  

                                                                                                                                                                                                                                                                                                                `|`                                                                                                                                                                                                                                                                                                              
 `aataagagacaggCGGCACCCATCGTTAGTAACGTACCGAGGAACCGAAGACAACGCTAAAGAGAAAATGTAAT‑‑CACACTGGGTCACCTTCGGGTGGGCCTTTCTGCGTTTATAAGGAGACACTTTATGTTTAAGAAGGTTGGTAAATACCTTGCGGCTTTGGCAGCTATCCTGACGCTTGCGTATATTCTTGAGGTATACCCTCAAGTAGCACTAGTAGTAGTGGGCGCTTGTTACTTAGCGGCAGTGTGTGCTTACGTGTGGAGTATAGTTAACTGGTAATACGACT‑‑CACTAAaggagg                                                                                                                                                                                                                                                                                                             <  1:761265/289‑1 (MQ=255)`  
        `tctTTGCGGCACCCGTCGTTCGTAATGTTCCGTGGCACCGAGGACAACCCTCAAGAGAAAATGTAAT‑‑CACACTGGCTCACCTTCGGGTGGGCCTTTCTGCGTTTATAAGGAGACACTTTATGTTTAAGAAGGTTGGTAAATTCCTTGCGGCTTTGGCAGCTATCCTGACGCTTGCGTATATTCTTGCGGTATACCCTCAAGTAGCACTAGTAGTAGTTGGCGCTTGTTACTTAGCTGCAGTGTGTGCTTGCGTGTGGAGTATAGTTAACTGGTAATACGACT‑‑CACTAAAGGAGGTacacac                                                                                                                                                                                                                                                                                                      <  1:390882/301‑1 (MQ=255)`  
                `gCACCCATCGTTCGTAATGTTCCGTGGCACCGAGGACAACCCTCAAGAGAAAATGTAAT‑‑CACACTGGCTCACCTTCGGGTGGGCCTTTCTGCGTTTATAAGGAGACACTTTATGTTTAAGAAGGTTGGTAAATTCCTTGCGGCTTTGGCAGCTATCCTGACGCTTGCGTATATTCTTGCGGTATACCCTCAAGTAGCACTAGTAGTAGTTGGCGCTTGTTACTTAGCGGCAGTGTGTGCTTGCGTGTGGAGTATAGTTAACTGGTAATACGACT‑‑CACTAAAGGAGGTACACACAATGATGt                                                                                                                                                                                                                                                                                              <  1:215947/301‑1 (MQ=255)`  
                `aaagagacagttcgtAATGTTCCGTGGCACCTAGGACAACCCTCAAGATAAAATGTAAT‑‑CACACTGGCTCACCTTCGGGTGGGCCTTTCTGCGTTTATAAGGAGACACTTTATGTTTAAGAAGGTTGGTAAATTCCTTGCGGCTTTGGCAGCTATCCTGACGCTTGCGTATATTCTTGCGGTATACCCTCAAGTAGCACTAGTAGTAGTTGGCGCTTGTTACTTAGCGGCAGTGTGTGCTTGCGTGTGGAGTATAGTTAACTGGTAATACGACT‑‑CACTAAAGGATGTACACACCATGATGt                                                                                                                                                                                                                                                                                              <  1:820708/292‑1 (MQ=255)`  
                  `ccccATCGTTCGTAATGTTCCGTGGCACCGAGGACAACCCTCAAGAGAAAATGTAAT‑‑CACACTGGCTCACCTTCGGGTGGGCCTTTCTGCGTTTATAAGGAGACACTTTATGTTTAAGAAGGTTGGTAAATTCCTTGCGGCTTTGGCAGCTATCCTGACGCTTGCGTATATTCTTGCGGTATACCCTCAAGTAGCACTAGTAGTAGTTGGCGCTTGTTACTTAGCGGCAGTGTGTGCTTGCGTGTGGAGTATAGTTAACTGGTAATACGACT‑‑CACTAAAGGAGGTACACACCATGATGTAc                                                                                                                                                                                                                                                                                            <  1:229677/300‑1 (MQ=255)`  
                    `ccATCGTTCGTAATGTTCCGTGGCACCGAGGACAACCCTCAAGAGAAAATGTAATCACACACTGGCTCACCTTCGGGTGTGCCTTTCTGCGTTTATAAGGAGACACTTTATGTTTAAGAAGGTTGGTAAATTCCTTGCGGCTTTGGCAGCTATCCTGACGCTTGCGTATATTCTTGCGGTATACCCTCAAGTAGCACTAGTAGTAGTTGGCGCTTGTTACTTAGCTGCAGTGTGTGCTTGCGTGTGGAGTATAGTTAACTGGTAATACGACT‑‑CACTAAAGGAGGTACACACCATGATGTAc                                                                                                                                                                                                                                                                                            <  1:405653/301‑1 (MQ=255)`  
                    `ccATCGTTCGTAATGTTCCGTGGCACCGAGGACAACCCTCAAGAGAAAATGTAAT‑‑CACACTGGCTCACCTTCTGGTGGGCCTTTCTGCGTTTATAAGGAGACACTTTATGTTTAAGAAGGTTGGTAAATTCCTTGCGGCTTTGGCAGCTATCCTGACGCTTGCGTATATTCTTGCGGTATACCCTCAAGTAGCACTAGTAGTAGTTGGCGCTTGTTACTTAGCGGCAGTGTGTGCTTGCGTGTGGAGTATAGTTAACTGGTAATACGACT‑‑CACTAAAGGAGGTACACACCATGATGTACtt                                                                                                                                                                                                                                                                                          <  1:285480/301‑1 (MQ=255)`  
                           `tcgtAATGTTCCGTGGCACCGAGGACAACCCTAAAGAGAAAATGTAAT‑‑CACACTGGCTCACCTTCGGGTGGGCCTTTCTGTGTTTATAAGGAGACACTTCATGTTTAAGAAGGTTGGTAAATTCCTTGCTGCTTTGGCAGCTATCCTGACGCTTGCGTATATTCTTGCGGTATACACTCATGTAGAACTCGTAGTCGTTGTCGCTTGTTACTTCGCGGCAGTGTGTGCTTGCGTGTGGAGTTTAGTTAACTGGTAATACTACT‑‑CACTTAATGATGTACATACCATTATTTACTTAATGCCa                                                                                                                                                                                                                                                                                   >  1:634825/1‑301 (MQ=255)`  
                                 `tGTTCCATGGTACCGAGGACAACCCTCAAGAGAAAATGTAAT‑‑CACACTGGCTCACCTTCGGGTGGGCCTTTCTGCTTTTATAAGGAGACACTTTATGTTTAAGAAGGTTGGTAAATTCCTTGCGGCTTTGGCAGCTATCCTTACGCTTGCGTATATTCTTGCGGTATACCCTCAAGTAGCACTAGTAGTAGTTGGCGCTTGTTACTTAGCGGCAGTGTGTGCTTGCGTGTGGAGTATAGTTAACTGGGAATACGACT‑‑CACTAAAGTAGGTACACACCATGAGGGAAGTAATGCAAGTACTc                                                                                                                                                                                                                                                                             <  1:807277/301‑1 (MQ=255)`  
                                             `ccGAGGACAACCCTCAAGAGAAAATGTAAT‑‑CACACTGGCTCACCTTCGGGTGGGCCTTTCTGCGTTTATAAGGAGACACTTTATGTTTAAGAAGGTTGGTAAATTCCTTGCGGCTTTGGCAGCTATCCTGACGCTTGCGTATATTCTTGCGGTATACCCTCAAGTAGCACTAGTAGTAGTTGGCGCTTGTTACTTAGCGGCAGTGTGTGCTTGCGTGTGCAGTATAGTTAACTGGTAATACGACT‑‑CACTAAAGGAGGTACACACCATGATGTACTTAATGCCATTACTCATCGTCATTGTa                                                                                                                                                                                                                                                                 >  1:197574/1‑301 (MQ=255)`  
                                                `aGGACAACCCTCAAGAGAAAATGTAAT‑‑CACACTGGCTCACCTTCGGGTGGGCCTTTCTGCGTTTATAAGGAGACACTTTATGTTTAAGAAGGTTGGTAAATTCCTTGCGGCTTTGGCAGCTATCCTGACGCTTGCGTATATTCTTGCGGTATACCCTCAAGTAGCACTAGTAGTAGTTGGCGCTTGTTACTTAGCGGCAGTGTGTGCTTGCGTGTGGAGTATAGTTAACTGGTAATACGACT‑‑CACTAAAGGAGGTACACACCATGATGTACTTAATGCCATTACTCATCGTTATTGTAGGa                                                                                                                                                                                                                                                              >  1:605595/1‑301 (MQ=255)`  
                                                      `aCCCTCAAGAGAAAATGTAAT‑‑CACACTGGCTCACCTTCGGGTGGGCCTTTCTGCGTTTATAAGGAGACACTTTATGTTTAAGAAGGTTGGTAAATTCCTTGCGGCTTTGGCAGCTATCCTGACGCTTGCGTATATTCTTGCGGTATACCCTCAAGTAGCACTAGTAGTAGTTGGCGCTTGTTACTTAGCGGCAGTGTGTGCTTGCGTGTGGAGTATAGTTAACTGGTAATACGACT‑‑CACTAAAGGAGGTACACACCATGATGTACTTAATGCCCTTACTCATCGTCATTGTAGGATGCCtt                                                                                                                                                                                                                                                        >  1:550467/1‑301 (MQ=255)`  
                                                               `agaAAATGTCAT‑‑CACACTGGCTCACCTTCGGGTTGGCCTTTCTGCGTTTATAAGGAGACACTTTATGTTTAAGAAGGTTGGTAAATTCCTTGCGGCTTTGGCAGCTATCCTGACGCTTGCGTATATTCTTGCTGTATACCCTCAAGTAGCACTAGTAGTAGTTGGCGCTTGTTACTTAGCGGCAGTGTGTGCTTGCGTGTGGAGTATAGTTAACTGGTAATACGACT‑‑CACTAAAGGAGGTATACACCATGATGTACTTAATGCCATTACTCATCGTCATTGTAGGATGCCTTGCGCTCAAc                                                                                                                                                                                                                                               <  1:563631/301‑1 (MQ=255)`  
                                                                   `aaTGTAAT‑‑CACACTGGCTCACCTTCGGGTGGGCCTTTCTGCGTTTATAAGGAGACACTTTATGTTTAAGAAGGTTGGTAAATTCCTTGCGGCTTTGGCAGCTATCCTGACGCTTGCGTATATTCTTGCGGTATACCCTCAAGTAGCACTAGTAGTAGTTGGCGCTTGTTACTTAGCGGCAGTGTGTGCTTGCGTGTGGAGTATAGTTAACTGGTAATACGACT‑‑CACTAAAGGAGGTACACACCATGATGTACTTAATGCCATTACTCATCGTCATTGTAGGATGCCTTGCGCTCCACTGtt                                                                                                                                                                                                                                           >  1:585636/1‑300 (MQ=255)`  
                                                                    `aagTAAT‑‑CACACTGGCTCACCTTCGGGTGGGCCTTTCTGCGTTTATAAGGAGACACTTTATGTTTAAGAAGGTGGGTAAATTCCTTGCGGCTTTGGCAGCTATCCTGACGCTTGCGTATATTCTTGCAGTATACCCTCAAGTAGCACTAGTAGTAGTTGGCGCTTGTTACTTAGCGGCAGTGTGTGCTTGCGTGTGGAGTATAGTTAACTGGTAATACGACT‑‑CACTAAAGGAGGTACACACCATGATGTACTTAATGCCATTACTCATCGTCATTGTAGGATGCCTTGCGCTCCACTGTAg                                                                                                                                                                                                                                          <  1:45629/299‑1 (MQ=255)`  
                                                                      `aagagacagcacTGGCTCACCTTCGGGTGGGCCTTTCTGCGTTTATAAGGAGACACTTTATGTTTAAGAAGGTTGGTAAATTCCTTGCGGCTTTGGCAGCTATCCTGACGCTTGCGTATATTCTTGCGGTATACCCTCAAGTAGCACTAGTAGTAGTTGGCGCTTGTTACTTAGCGGCAGTGTGTGCTTGCGTGTGGAGTATAGTTAACTGGTAATACGACT‑‑CACTAAAGGAGGTACACACAATGATGTACTTAATGCCATTACTCATCGTCATTGTAGGATGCCTTGCGCTCCACTGTAg                                                                                                                                                                                                                                          <  1:112898/292‑1 (MQ=255)`  
                                                                         `aT‑‑CACACTGGCTCACCTTCGGGTGGGCCTTTCTGCGTTTATAAGGAGACACTTTATGTTTAAGAAGGTTGGTAAATTCCTTGCGGCTTTGGCAGCTATCCTGACGCTTGCGTATATTCTTGCGGTATACCATCAAGTAGCACTAGTAGTAGTTGGCGCTTGTTACTTAGCGGCAGTGTGTGCTTGCGTGTGGAGTATAGTTAACTGGTAATACGACT‑‑CACTAAAGGAGGTACACACCATGATGTACTTAATGCCATTACTCATCGTCATTGTAGGATGCCTTGCGCTCCACTGTAGCgatg                                                                                                                                                                                                                                     <  1:272079/301‑1 (MQ=255)`  
                                                                             `cacaCTGGCTCACCTTCGGGTGGGCCTTTCTGCGTTTATAAGGAGACACTTTATGTTTAAGAAGGTTGGTAAATTCCTTGCGGCTTTGGCAGCTATCCTGACGCTTGCGTATATTCTTGCGGTATACCCTCAAGTAGCACTAGTAGTAGTTGGCGCTTGTTACTTAGCGGCAGTGTGTGCTTGCGTGTGGAGTATAGTTAACTGGTAATACGACT‑‑CACTAAAGGAGGTACACACCATGATGTACTTAATGCCATTACTCATCGTCATTGTAGGATGCCTTGCGCTCCACTGTAGCgatgat                                                                                                                                                                                                                                   >  1:667758/1‑301 (MQ=255)`  
                                                                               `taagagacagccTTCGGGTGGGCCTTTCTGCGTTTATAAGGAGACACTTTATGTTTAAGAAGGTTGGTAAATTCCTTGCGGCTTTGGCAGCTATCCTGACGCTTGCGTATATTCTTGTGGTATACCCTCAAGTAGCACTAGTAGTAGTTGGCGCTTGTTACTTAGCGGCAGTGTGTGCTTGCGTGTGGAGTATAGTTAACTGGTAATACGACT‑‑CACTAAAGGAGGTACACACCATGATGTACTTAATGCCATTACTCATCGTCATTGTAGGATGCCTTGCGCTCCACTGTAGCgatgatga                                                                                                                                                                                                                                 <  1:384270/291‑1 (MQ=255)`  
                                                                               `cacTGGCTCACCTTCGGGTGGGCCTTTCTGCGTTTATAAGGAGACACTTTATGTTTAAGAAGGTTGGTAAATTCCTTGCGGCTTTGGCAGCTATCCTGACGCTTGCGTATATTCTTGCGGTATACCCTCAAGTAGCACTAGTAGTAGTTGGCGCTTGTTACTTAGCGGCAGTGTGTGCTTGCGTGTGGAGTATAGTTAACTGGTAATACGACT‑‑CACTAAAGGAGGTATAAACCATGATGTACTTAATGCCATTACTCATCGTCATTGTAGGAGGCCTTGCACTCCAcctgtatctgataca                                                                                                                                                                                                                                 >  1:2385/1‑287 (MQ=255)`  
                                                                               `cacTGGCTCACCTTCGGGTGGGCCTTTCTGCGTTTATAAGGAGACACTTTATGTTTAAGAAGGTTGGTAAATTCCTTGCGGCTTTGGCAGCTATCCTGACGCTTGCGTATATTCTTGCGGTATACCCTCAAGTAGCACTAGTAGTAGTTGGCGCTTGTTACTTAGCGGCAGTGTGTGCTTGCGTGTGGAGTATAGTTAACTGGTAATACGACT‑‑CACTAAAGGAGGTACACACCATGATGTACTTAATGCCATTACTCATCGTCATTGTAGGATGCCTTGCGCTCCACTGTAGCgatgatga                                                                                                                                                                                                                                 >  1:206137/1‑301 (MQ=255)`  
                                                                               `cacTGGCTCACCTTCGGGTGGGCCTTTCTGCGTTTATAAGGAGACACTTTATGTTTAAGAAGGTTGGTAAATTCCTTGCGGCTTTGGCAGCTATCCTGACGCTTGCGTATATTCTTGCGGTATACCCTCAAGTAGCACTAGTAGTAGTTGGCGCTTGTTACTTAGCGGCAGTGTGTGCTTGCGTGCGGAGTATAGTTAACTGGTAATACGACT‑‑CACTAAAGGAGGTACACACCATGATGTACTTAATGCCATTACTCATCGTCGTTGTAGGATGCCTTGCGCTCCACTGTAGCgatgatga                                                                                                                                                                                                                                 >  1:265456/1‑301 (MQ=255)`  
                                                                               `cacTGGCTCACCTTCGGGTGGGCCTTTCTGCGTTTATAAGGAGACACTTTATGTTTAAGAAGGTTGGTAAATTCCTTGCGGCTTTGGCAGCTATCCTGACGCTTGCGTATATTCTTGCGGTATACCCTAAAGTAGCACTAGTAGTAGTTGGCGCTTGTTACTTAGCGGCAGTGTGTGCTTGCGTGTGGAGTATAGTTAACTGGTAATACGACT‑‑CACTAAAGGAGGTACACACCATGATGTACTTAATGCCATTACTCATCGTCATTGTAGGATGCCTTGCGCTCCAcctgtctctatacac                                                                                                                                                                                                                                 >  1:618440/1‑287 (MQ=255)`  
                                                                                 `cTGGCTCACCTTCGGGTTGGCCTTTCTGCGTTTATAAGGAGACACTTTATGTTTAAGAAGGTTGGTAAATTCCTTGCGGCTTTGGCAGCTATCCTGACGCTTGCGTATATTCTTGCGGTATACCCTCAAGTAGCACTGGTAGTAGTTGGCGCTTGTTACTTAGCGGCAGTGTGTGCTTGCGTGTGGAGTATAGTTAACTGGTAATACGACT‑‑CACTAAAGGAGGTACACACCATGATGTACTTAATGCCATTACTCATCGTCATTGTAGGATGCCTTGCGCTCCACTGTAGCgatgatgatt                                                                                                                                                                                                                               >  1:332980/1‑300 (MQ=255)`  
                                                                                     `ggctcggagatgtgtataagagacagTTTATAAGGAGACACTTTATGTTTAAGAAGGTTGGTAAATTCCTTGCGGCTTTGGCAGCTATCCTGACGCTTGCGTATATTCTTGCGGTATACCATCAAGTAGCACTAGTAGTAGTTGGCGCTTGTTACTTAGCGGCAGTGTGTGCTTGCGTGTGGAGTATAGTTAACTGGTAATACGACT‑‑CACTAAAGGAGGTACACACCATGATGTACTTAATGCCATTACTCATCGTCATTGTAGAATGCCTTGCGCTCCACTGTAGCGATGATGATATGcc                                                                                                                                                                                                                           <  1:367417/276‑1 (MQ=255)`  
                                                                                              `gggTGGGCCTTTCTGCGTTTATAAGGAGACACTTTATGTTTAAGAAGGTTGGTAAATTCCTTGCGGCTTTGGCAGCTATCCTGACGCTTGCGTATATTCTTGCGGTATACCCTCAAGTAGCACTAGTAGTAGTTGGCGCTTGTTACTTAGCGGCAGTGTGTGCTTGCGTGTGGAGTATAGTTAACTGGTAATACGACT‑‑CACTAAAGGAGGTACACACCATGATGTACTTAATGCCATTACTCATCGTCATTGTAGGATGCCTTGCGCTCCACTGTAGCGATGATGATATGCCAGATGGTcc                                                                                                                                                                                                                  >  1:571208/1‑300 (MQ=255)`  
                                                                                                `gTGGGCCTTTCTGCGTTTATAAGGAGACACTTTATGTTTAAGAAGGTTGGTAAATTCCTTGCGGCGTTGGCAGCTATCCTGACGCTTGCGTATATTATTGCAGTATACCCTCAAGTAGCACTAGTAGTAGTTGGCGCTTGTTACTTAGCGGCAGTGTGTGCTTGCGGGTGGAGTATAGTTAACTGGTAATACGACT‑‑CACTAAAGGAGGTACACACCATGATGTACTTAATGCCATTACTCATCGTCATTGTAGGATGCCTTGCGCTCCACTGTAGCGATGATGATATGCCAGATGGTCACg                                                                                                                                                                                                                >  1:119481/1‑301 (MQ=255)`  
                                                                                                        `ttCTGCGTTTATAAGGAGACACTTTATGTTTAAGAAGGTTGGTAAATTCCTTGCGGCTTTGGCAGCTATCCTGACGCTTGCGTATATTCTTGCGGTATACCCTCAAGTAGCACTAGTAGTAGTTGGCGCTTGTTACTTAGCGGCAGTGTGTGCTTGCGTGTGGAGTATAGTTAACTGGTAATACGACT‑‑CACTAAAGGAGGTACACACCATGATGTACTTAATGCCATTACTCATCGTCATTGTAGGATGCCTTGCGCTTCACTGTAGCGATGATGATATGCCAGATGGTCACGCTTAATAc                                                                                                                                                                                                        <  1:188511/301‑1 (MQ=255)`  
                                                                                                        `ttCTGCGTTTATAAGGAGAAACTTTATGTTTAAGAAGGTTGGTAAATTCCTTGCGGCTTTGGCAGCTATCCTGACGCTTACGTATATTCTTGCGGTATACCCTCAAGTAGCACTAGTAGTAGTTGGCGCTTGTTACTTAGCGGCAGTGTGTGCTTGCGTGTGGAGTATAGTTAACTGGTAATACGACT‑‑CACTAAAGGAGGTACACACCATGATGTACTTAATGCCATTACTCATCGTCATTGTAGGATGCCTTGCGCTCCACTGTAGCGATGATGATATGCCAGATGGTCACGCTTAATAc                                                                                                                                                                                                        <  1:234063/301‑1 (MQ=255)`  
                                                                                                           `tGCGTTTATAAGGAGACACTTTATGTTTAAGAAGGTTGGTAAATTCCTTGCGGCTTTGGCAGCTATCCTGACGCTTGCGTATATTCTTGCGGTATACCCTCAAGTAGCACTAGTAGTAGTTGGCGCTTGTTACTTAGCGGCAGTGTGTGCTTGCGTGTGGAGTATAGTTAACTGGTAATACGACT‑‑CACTAAAGGAGGTACACACCATGATGTACTTAATGCCATTACTCATCGTCATTGTAGGATGCCTTGCGCTCCACTGTAGCGATGATGATATGCCAGATGGTCACGCTTAATACGAc                                                                                                                                                                                                     <  1:222522/301‑1 (MQ=255)`  
                                                                                                              `gTTTATAAGGAGACACTTTATGTTTAAGAAGGTTGGTAAATTACTTGCGGCTTTGGCAGCTATCCTGACGCTTGCGTATATTCTTGCGGTATACCCTCAAGTAGCACTAGTAGTAGTTGGCGCTTGTTACTTAGCGGCAGTGTGTGCTTGCGTGTGGAGTATAGTTAACTGGTAATACGACT‑‑CACTAAAGGAGGTACACACCATGATGTACTTAATGCCATTACTCATCGTCATTGTAGGATGCCTTGCGCTCCACTGTAGCGATGATGATATGCCAGATGGTCACGCTTAATACGACTCa                                                                                                                                                                                                  >  1:529420/1‑301 (MQ=255)`  
                                                                                                                `ttATAAGGAGACACTTTATGTTTAAGAAGGTTGGTAAATTCCTTGCGGCTTTGGCAGCTATCCTGACGCTTGCGTATATTCTTGCGGTATACCCTCAAGTAGCACTAGTAGTAGTTGGCGCTTGTTACTTAGCGGCAGTGTGTGCTTGCGTGTGGAGTATAGTTAACTGGTAATACGACT‑‑CACTAAAGGAGGTACACACCATGATGTACTTAATGCCATTACTCATCGTTATTGTAGGATGCCTTGCGCTCCACTGTAGCGATGATGATATGCCAGATTGTCACGCTTAATACGACTCACt                                                                                                                                                                                                <  1:709875/301‑1 (MQ=255)`  
                                                                                                                     `tgtgtataagagacagttAAGAAGGTTGGTAAATTCCTTGCGGCTTTGGCAGCTATCCTGACGCTTGCGTATATTCTTGCGGTATACCCTCAAGTAGCACTAGTAGTAGTTGGCGCTTGTTACTTAGCGGCAGTGTGTGCTTGCGTGTGGAGTATAGTTAACTGGTAATACGACT‑‑CACTAAAGGAGGTACACACCATGATGTACTTAATGCCATTACTCATCGTCATTGTAGGATGCCTTGCGCTCCACTGTAGCGATGATGATATGCCAGATGGTCACGCTTAATACGACTCACTAAAgg                                                                                                                                                                                           <  1:249531/285‑1 (MQ=255)`  
                                                                                                                     `aGGAGACACTTTATGTTTAAGAAGGTTGGTAAATTCCTTGCGGCTTTGGCAGCTATCCTGACGCTTGCGTATATTATTGCGGTATACCCTCAAGTAGCACTAGTAGTAGTTGGCGCTTGTTACTTAGCGGCAGTGTGTGCTTGCGTGTGGAGTATAGTTAACTGGTAATACGACT‑‑CACTAAAGGAGGTACACACCATGATGTACTTAATGCCATTACTCATCGTCATTGTAGGATGCCTTGCGCTCCACTGTAGCGATGATGATATGCCAGATGGTCACGCTTAATACGACTCACTAAAgg                                                                                                                                                                                           <  1:780668/301‑1 (MQ=255)`  
                                                                                                                       `gagaCACTTTATGTTTAAGAAGGTTGGTAAATTCCTTGCGGCTTTGGCAGCTATCCTGACGCTTGCGTATATTCTTGCGGTATACCCTCATGTAGCACTAGTAGTAGTTGGCGCTTGTTACTTAGCGGCAGTGTGTGCTTGCGTGTGGAGTATAGTTAACTGGTAATACGACT‑‑CACTAAAGGAGGTACACACCATGATGTACTTAATGCCATTACTCATCGTCATTGTAGGATGCCTTGCGATCCACTGTAGCGATGATGATATGCCAGATGGTCACGCTTAATACGACTCACTAAAGgag                                                                                                                                                                                         >  1:87336/1‑301 (MQ=255)`  
                                                                                                                       `gagaCACTTTATGTTTAAGAAGGTTGGTAAATTCCTTGCGGCTTTGGCAGCTATCCTGACGCTTGCGTATATTCTTGCGGTATACCCTCAAGTAGCACTAGTAGTAGTTGGCGCTTGTTACTTAGCGGCAGTGTGTGCTTGCGTGTGGAGTATAGTTAACTGGTAATACGACT‑‑CACTAAAGGAGGTACACACCATGATGTACTTAATGCCATTACTCATCGTCATTGTAGGATGCCTTGCGCTCCACTGTAGCGATGATGATATGCCAGATGGTCACGCTTAATACGACTCACTAAAGgag                                                                                                                                                                                         <  1:344609/301‑1 (MQ=255)`  
                                                                                                                       `gagaCACTTTATGTTTAAGAAGGTTGGTAAATTCCTTGCGGCTTTGGCAGCTATCCTGACGCTTGCGTATATTCTTGCGGTATACCCTAAAGTAGCAATAGTAGTAGTTGGCGCTTGTTACTTAGCGGCAGTGTGTGCTTGCGTGTGGAGTATAGTTAACTGGTAATACGACT‑‑CACTAAAGGAGGTACACACCATGATGTACTTAATGCCATTACTAATCGTCATTGTAGGATGCCTTGCGCTCCACTGTAGCGATGATGATATGCCAGATGGTCACGCTTAATACGACTCACTAAAGgag                                                                                                                                                                                         >  1:612510/1‑301 (MQ=255)`  
                                                                                                                          `acagTTTAGGTTTAAGAAGGTTGGTAAAGTCCTTGCGGCGTTGGCAGTTATCCTGACGCTTGCGTATATTCTTGCGGTAGACCGCCAAGTAGCACTAGTAGTAGTTGGCGCTTGTTACTTAGCGGCAGTGTGTGCTTGCGTGTGGAGTATAGTTAACTGGTAATACGACT‑‑CACTAAAGGAGGTACACACTATGATGTACTTAATGCCATTGCTCATCGTCATTGTAGGATGCCTTGCGCTCCACTGGAGGGATGAGGATATGCCAGATGGTCACGCTTAATACGACTCACTAAAAGAGacc                                                                                                                                                                                      <  1:687110/301‑2 (MQ=255)`  
                                                                                                                                 `gtgtataagagacagggTAAATTCCTTGCGGCTTTGGCAGCTATCCTGACGCTTGCGTATATTCTTGCGGTATACCCTCAAGTAGCACTAGTAGTAGTTGGCGCTTGTTACTTAGCGGCAGTGTGTGCTTGCGTGTGGAGTATAGTTAACTGGTAATACAACT‑‑CACTAAAGGAGGTACACACCATGATGTACTTAATGCCATTACTCATCGTCATTGTAGGATGCCTTGCGCTCCACTGTAGCGATGATGATATGCCAGATGGTCACGCTTAATACGACTCACTAAAGGAGACACTATATg                                                                                                                                                                               <  1:145178/286‑1 (MQ=255)`  
                                                                                                                                 `aTGTTTAAGAAGGTTGGTAAATTCCTTGCGGCTTTGGCAGCTATCCTGACGCTTGCGTATATTCTTGCGGTATACCCTCAAGTAGCACTAGTAGTAGTTGGCGCTTGTTACTTAGCGGCAGTGTGTGCTTGCGTGTGGAGTATAGTTAACTGGTAATACGACT‑‑CACTAAAGGAGGTACACACCATGATGTACTTAATGCCATTACTCATCGTCATTGTAAGATGCCTTGCGCTCCACTGTAGCGATGATGATATGCCAGATGGTCACGCTTAATACGACTCACTAAAGGAGACACTATATg                                                                                                                                                                               >  1:732222/1‑301 (MQ=255)`  
                                                                                                                                   `gtataagagacagggTAAATTCCTTGCGGCTTTGGCAGCTATCCTGACGCTTGCGTATATTCTTGCGGTATACCCTCAAGTAGCACTAGTAGTAGTTGGCGCTTGTTACTTAGCGGCAGTGTGTGCTTGCGTGTGGAGTATAGTTAACTGGTAATACGACT‑‑CACTAAAGGAGGTACACACCATGATGTACTTAATGCCATTACTCATCGTCATTGTAGGATGCCTTGCGCTCCACTGTAGCGATGATGATATTCCAGATGGTCACGCTTAATACGACTCACTAAAGGAGACACTATATGtt                                                                                                                                                                             <  1:503771/288‑1 (MQ=255)`  
                                                                                                                                        `agaagGTTGGTAAATTCCTTGCGGCTTTGGCAGCTATCCTGACGCTTGCGTATATTCTTGCGGTATACCCTCAAGTAGCACTAGTAGTAGTTGGCGCTTGTTACTTAGCGGCAGTGTGTGCTTGCGTGTGGAGTATAGTTAACTGGTAATACGACT‑‑CACTAAAGGAGGTACACACCATGATGTACTTAATGCCATTACTCATCGTCATTGTAGGATGCCTTGCGCTCCACTGTAGCGATGATGATATGCCAGATGGTCACGCTTAATACGACTCACTAAAGGAGACACTATATGTTTCGAc                                                                                                                                                                        <  1:49643/301‑1 (MQ=255)`  
                                                                                                                                                `ggTAAATTCCTTGCGGCTTTGGCAGCTATCCTGACGCTTGCGTATATTCTTGCGGTATACCCTCAAGTAGCACTAGTAGTAGTTGGAGCTTGTTACTTAGCGGCAGTGTGTGCTTGCGTGTGGAGTATAGTTAACTGGTAATACGACT‑‑CACTAAAGGAGGTACACACCATGATGTACTTAATGCCATTACTCATCGTCATTGTAGGATGCCTTGCGCTCCACTGTAGCGATGATGATATGCCAGATGGTCACGCTTAATACGACTCACTAAAGGAGACACTATATGTTTCGACTTCATTac                                                                                                                                                                >  1:276091/1‑301 (MQ=255)`  
                                                                                                                                                                         `cTATCCTGACGCTTGCGTATATTCTTGCGGTATACCCTCAAGTAGCACTAGTAGTAGTTGGCGCTTGTTACTTAGCGGCAGTGTGTGCTTGCGTGCGGAGTATAGTTAACTGGTAATACGACT‑‑CACTAAAGGAGGTACACACCATGATGTACTTAATGCCATTACTCATCGTCATTGTAGGATGCCTTGCGCTCCACTGTAGCGATGATGATATGCCAGATGGACACGCTTAATACGACTCACTAAAGGAGAAACTATATGTTTCGACTTCATTACAACAAAAGCGTTAAGAATTTCACgg                                                                                                                                       <  1:94107/301‑1 (MQ=255)`  
                                                                                                                                                                           `aTCCTGACGCTTGCGTATATTCTTGCGGTATACCCTAAAGTAGCACTAGTAGTAGTTGGCGCTTGTTACTTAGCGGCAGTGTGTGCTTGCGTGTGGAGTATAGTTAACTGGTAATACGACT‑‑CACTAAAGGAGGTACACACCATGATGTACTTAATGCCATTACTCATCGTCATTGTAGGATGCCTTGCGCTCCACTGTAGCGATGATGATATGCCAGATGTTCACGCTTAATACGACTCACTAAAGGAGACACTATATGTTTCAACTTCATTACAACAAAAGCGTTAAGAATTTCACGGtt                                                                                                                                     >  1:190344/1‑301 (MQ=255)`  
                                                                                                                                                                                   `gCTTGCGTATATTCTTGCTGTATATCCTCAGGTAGCACTAGTAGTAGTGGGGGCTTGTTACTTAGCGGCAGTGTGGGCTTTCGTGTGGAGTATAGTTAACTGGTAATACGACT‑‑CACTAAAGGAGGTACACACCATGATGTACTTAATGCCATTACTCATCGTCATTGTAGGATGCCTTGCGCTCCACTGTAGCGATGATGATATGCTAGATGGTCACGCTTAATACGACTCACTAAAGGAGACACTATATGTTTCGACTTCATTACAACAAAAGCGTTAATAATTTCACGGTTCGCCGTGc                                                                                                                             <  1:789352/301‑1 (MQ=255)`  
                                                                                                                                                                                    `cTTGCGTATATTCTTGCGGTATACCCTCAAGTAGCACTAGTAGTAGTTGGCGCTTGTTACTTAGCGGCAGTGTGTGCTTGCGTGTGGAGTATAGTTAACTGGTAATACGACT‑‑CACTAAAGGAGGTACACACCATGATGTACTTAATGCCATTACTCATCGTCATTGTAGGATGCCTTGCGCTCCACTGTAGCGATGATGATATGCCAGATGGTCACGCTTAATACGACTCACTAAAGGAGACACTATATGTTTCGACTTCATTACAACAAAAGCGTTAAGAATTTCAcctgtctcttatac                                                                                                                            >  1:594570/1‑288 (MQ=255)`  
                                                                                                                                                                                     `ttGCGTATATTCTTGCGGTATACCCTCAAGTAGCACTAGTAGTAGTTGGTGCTTGTTACTTAGCGGCAGTGTGTGCTTGCGTGTTGAGTATAGTTAACTGGTAATACGACT‑‑CACTAAAGGAGGTACACACCATGATGTACTTAATGCCATTACTCATCGTCATTGTAGGATGCCTTGCGCTCCACTGTAGCGATGATGATATGCCAGATGGTCACGCTTAATACGACTCACTAAAGGAGACACTATATGTTTCGACTTCATTACAACAAAAGCGTTAAGAATTTCACGGTTCGCCGTGCTg                                                                                                                           <  1:657218/301‑1 (MQ=255)`  
                                                                                                                                                                                       `gagacagttCTTGCGGTATACCCTCAAGTAGCACTAGTAGTAGTTGGCGCTTGTTACTTAGCGGCAGTGTGTGCTTGCGTGTGGAGTATAGTTAACTGGTAATACGACT‑‑CACTAAAGGAGGTACACACCATGATGTACTTAATGCCATTACTCATCGTCATTGTAGGATGCCTTGCGCTCCACTGTAGCGATGATGATATGCCAGATGGTCACGCTTAATACGACTCACTAAAGGAGACACTATATGTTTCGACTTCATTACAACAAAAGCGTTAAGAATTTCACGGTTCGCCGTGCTGAc                                                                                                                         <  1:25845/294‑1 (MQ=255)`  
                                                                                                                                                                                                `cTTGCGGTATACCCTCAAGTATCACTAGTAGTAGTTGGCGCTTGTTACTTAGCGGCAGTGTGTGCTTGCGTGTGGAGTATAGTTAACTGGTAATACGACT‑‑CACTAAAGGAGGTACACACCATGATGTACTTAATGCCATTACTCATCGTCATTGTAGGATGCCTTGCGCTCCACTGTAGCGATGATGATATGCAAGATGGTCACGCTTAATACGACTCACTAAAGGAGACACTATATGTTTCGACTTCATTACAACAAAAGCGTTAAGAATTTCACGGTTCGCCGTGCTGACCATTCAATc                                                                                                                >  1:518613/1‑301 (MQ=255)`  
                                                                                                                                                                                                 `ttGCGGTATACCCTAAAGTAGCACTAGTAGTAGTTGGCGCTTGTTACTTAGCGGCAGTGTGTGCTTGCGTGTGGAGTATAGTTAACTGGTAATACGACT‑‑CACTAAAGGAGGTACACACCATGATGTACTTAATGCCATTACTCATCGTCATTGTAGGATGCCTTGCGCTCCACTGTAGCGATGATGATATGCCAGATGGTCACGCTTAATACGACTCACTAAAGGAGACACTATATGTTTCGACTTCATTACAACAAAAGCGTTAAGAATTTCACGGTTCGCCGTGCTGACCGTTCAATCg                                                                                                               <  1:793713/301‑1 (MQ=255)`  
                                                                                                                                                                                                     `tataagagtcagaGTAGCACTAGTAGTAGTTGGCGCTTGTTACTTAGCGGCAGTGTGTGCTTGCGTGCGGAGTATAGTTAACTGGTAATACGACT‑‑CACTAAAGGAGGTACACACCATGATGTACTTAATGCCATTACTCATCGTCATTGTAGGATGCCTTGCGCTCCACTGTAGCGATGATGATATGCCAGATGGTCACGCTTAATACTACTCACTAAAGGAGACACTATATGTTTCGACTTCATTACAACAAAAGCGTTAAGAATTTCACGGTTCGCCGTGCTGACCGTTCAATCGTAtg                                                                                                           <  1:699653/289‑1 (MQ=255)`  
                                                                                                                                                                                                           `cccTCAAGTAGCACTAGTAGTAGTTGGCGCTTGTTACTTAGCGGCAGTGTGTGCTTGCGTGTGGAGTATAGTTAACTGGTAATACGACT‑‑CACTAAAGGAGGTACACACCATGATGTACTTAATGCCATTACTCATCGTCATTGTAGGATGCCTTGCGCTCCACTGTAGCGATGATGATATGCCAGATGGTCACGCTTAATACGACTCACTAAATGAGACACTATATGTTTCGACTTCATTACAACAAAAGCGTTAAGAATTTCACGGTTCGCCGTGCTGACCGTTCAATCGTATGTgcgag                                                                                                     >  1:134342/1‑301 (MQ=255)`  
                                                                                                                                                                                                           `cccTCAAGTAGCACTAGTAGTAGTTGGCGCTTGTTACTTAGCGGCAGTGTGTGCTTGCGTGTGGAGTATAGTTAACTGGTAATACGACT‑‑CACTAAAGGAGGTACACACCATGATGTACTTAATGCCATTACTCATCGTCATTGTAGGATGCCTTGCGCTCCACTGTAGCGATGATGATATGCCAGATGGTCACGCTTAATACGACTCACTAAAGGAGACACTATATGTTTCGACTTCATTACAACAAAAGCGTTAAGAATTTCACGGTTCGCCGTGCTGACCGTTCAATCGTATGTgcgag                                                                                                     >  1:4637/1‑301 (MQ=255)`  
                                                                                                                                                                                                             `cTCAAGTAGCACTAGTAGTAGTTGGCGCTTGTTACTTAGCGGCAGTGTGTGCTTGCGTGTGGAGTATAGTTAACTGGTAATACGACT‑‑CACTAAAGGAGGTACACACAATGATGTACTTAATGCCATTACTCATCGTCATTGTAGGATGCCTTGCGCTCCACTGTAGCGATGATGATATGCCAGATGGTCACGCTTAATACGACTCACTAAAGGAGACACTATATGTTTCGACTTCATTACAACAAAAGCGTTAAGAATTTCACGGTTCGCCGTGCTGACCGTTCAATCGTATGTgcgagcg                                                                                                   <  1:430763/301‑1 (MQ=255)`  
                                                                                                                                                                                                             `atgtgtataagagacaggtagtTGGCGCTTGTTACTTAGCGGCAGTGTGTGCTTGCGTGTGGAGTATAGTTAACTGGTAATACGACT‑‑CACTAAAGGAGGTACACACCATGATGTACTTAATGCCATTACTCATCGTCATTGTAGGATGCCTTGCGCTCCACTGTAGCGATGATGATATGCCAGATGGTCACGCTTAATACGACTCACTAAAGGAGACACTATATGTTTCGACTTCATTACAACAAAAGCGTTAAGAATTTCACGGTTCGCCGTGCTGACCGTTCAATCGTATGTgcgagcg                                                                                                   <  1:442309/284‑1 (MQ=255)`  
                                                                                                                                                                                                                  `cggagatgtgtataagagacagcTTGTTACTTAGCGGCAGTGTGTGCTTGCGTGTGGAGTATAGTTAACTGGTAATACGACT‑‑CACTAAAGGAGGTACACACCATGATGTACTTAATGCCATTACTCATCGTCATTGTAGGATGCCTTGCGCTCCACTGTAGCGATGATGATATGCCAGATGGTCACGCTTAATACGACTCACTAAAGGAGACACTATATGTTTCGACTTCATTACAACAAAAGCGTTAAGAATTTCACGGTTCGCCGTGCTGACCGTTCAATCGTATGTGCGAGCAAgcgc                                                                                              <  1:447498/280‑1 (MQ=255)`  
                                                                                                                                                                                                                         `tagtagtagTTGGCGCTTGTTACTTAGCGGCAGTGTGTGCTTGCGTGCGGAGTATAGTTAACTGGTAATACGACT‑‑CACTAAAGGAGGTACACACCATGATGTACTTAATGCCATTACTCATCGTCATTGTAGGATGCCTTGCGCTCCACTGTAGCGATGATGATATGCCAGATGGTCACGCTTAATACGACTCACTAAAGGAGACACTATATGTTTCGACTTCATTACAACAAAAGCGTTAAGAATTTCACGGTTCGCCGTGCTGACCATTAAATCGTATGTGCGAGCGAGCGCCGAGCTa                                                                                       >  1:749261/1‑301 (MQ=255)`  
                                                                                                                                                                                                                          `agtagtagtTGGCGCTTGTTACTTAGCGGCAGTGTGTGCTTGCGTGTGGAGTATAGTTAACTGGTAATACGACT‑‑CACTAAAGGAGGTACACACCATGATGTACTTAATGCCATTACTCATCGTCATTGTAGGATGCCTTGCGCTCCACTGTAGCGATGATGATATGCCAGATGGTCACGCTTAATACGACTCACTAAAGGAGACACTATATGTTTCGACTTCATTACAACCAAAGCGTTAAGAATTTCACGGTTCGCCGTGCTGACCATTCaactgtctcttatacacatctccgatcccc                                                                                      >  1:369933/1‑273 (MQ=255)`  
                                                                                                                                                                                                                            `aaagagacagcgcTTGTTACTTGGCGGCAGTGTGTGCTTGCGTGTGGAGTATAGTTAACTGGTAATACGACT‑‑CACTAAAGGAGGTACACACCATGATGTACTTAATGCCATTACTCATCGTCATTGTAGGATGCCTTGCGCTCCACTGTAGCGATGATGATATGCCAGATGGTCACGCTTAATACGACTCACTAAAGGAGACACTATATGTTTCGACTTCATTACAACAAAAGCGTTAAGAATTTCACGGTTCGCCGTGCTGACCGTTCAATCGTATGTGCGAGCGAGCGCCGAGCTAAGa                                                                                    <  1:649474/292‑1 (MQ=255)`  
                                                                                                                                                                                                                               `tagtTTGCGCTTGTTACTTAGCGGCAGTGTGTGCTTGCGTGTGGAGTATAGTTAACTGGTAATACGACT‑‑CACTAAAGGAGGTACACACCATGATGTACTTAATGCAATTACTCATCGTCATTGTAGGATGCCTTGCGCTCCACTGTAGCGATGATGATATGCCAGATGGTCACGCTTAATACGACTCACTAAAGGAGACACTATATGTTTCGACTTCATTACAACAAAAGCGTTAAGAATTTCACGGTTAGCCGTGCTGACCGTTCAATCGTATGTGCGAGCGAGCGCCGAGCTAAGATAc                                                                                 <  1:252847/301‑1 (MQ=255)`  
                                                                                                                                                                                                                                  `ttGGCGCTTGTTACTTAGCGGCAGTGTGTGCTTGCGTGTGGAGTATAGTTAACTGGTAATACGACT‑‑CACTAAAGGAGGTACACACCATGATGTACTTAATGCCATTACTCATCGTCATTGTAGGATGCCTTGCGCTCCACTGTAGCGATGATGATATGCCAGATGGTCACGCTTAATACGACTCACTAAAGGAGACACTATATGTTTCGACTTAATTACAACAAAAGCGTTAAGAATTTCACGGTTCGCCGTGCTGACCGTTCAATCGTATGTGCGAGCGAGCGCCGAGCTAAGATACctc                                                                              <  1:772870/301‑1 (MQ=255)`  
                                                                                                                                                                                                                                  `tgggCGATTGTTAAGTAGAGGCAGTGTGTGCTTGCGTGTGGAGTATAGTGAACTGGTAATACGACT‑‑CACTAAAGGAGGTACACACCATGATGTACTTAATGCCATTACTCATCGTCATTGTAGGATGCCTTGCGCTCCACTGTAGCGATGATGATATGCCAGATGGTCCCGCTTAATACGACTCACTAAAGGAGACACTATATGTTTCGACTTCATTACAACAAAAGCGTTAAGAATTTCACGGTTCGCCGTGCTGACCGTTCAATCGTATGTGCGAGCGAGCGCCGAGCTAAGATACctc                                                                              <  1:812921/299‑1 (MQ=255)`  
                                                                                                                                                                                                                                  `aatgggtagagagacaggCGGCAGTGGGTGCTTGCATGTGGAGTATAGTTAACTGGTAATACGACT‑‑CACTAAAGGAGGTACACACCATGATGTACTTAATGCCATTACTCATCGTCATTGTAGGATGCCTTGCGCTCCACTGTAGCGATGATGATATGCCAGATGGTCACGCTTAATACGACTCACTAAAGGAGACACTATATGTTTCGACTTCATTACAACAAAAGCGTTAAGAATTTCACGGTTCGCCGTGCTGACCGTTCAATCGTATGTGCGAGCGAGCGCCGAGCTAAGATACctc                                                                              <  1:137067/284‑1 (MQ=255)`  
                                                                                                                                                                                                                                        `gttGTTACTTAGCGGCAGTGTGTGCTTGCGTGTGGAGTATAGTTAACTGGTAATACGACT‑‑CACTAAAGGGGGTACACACCATGATGTACTTAATGCCATTACTCATCGTCATTGTAGGATGCATTGCGCTCCACTGTAGCGATGATGATATGCCAGATGGTCACGCTTAATACGACTCACTAAAGGAGACACTATATGTTTCGACTTCATTACAACAAAAGCGTTAAGAATTTCACAGTTCGCCGTGCTGACCGTTCAATCGTATGTGCGAGCGAGCGCCGAGCTAAGATACCTCTTATTg                                                                        <  1:740849/300‑1 (MQ=255)`  
                                                                                                                                                                                                                                          `tGTTACTTAGCGGCAGTGTTAGCTTGCGTGTGTAGTATAGTTAACTAGTAATACGACT‑‑CACTAAAGGAGGTACACACCATGATGTACTTAATGCCATTACTCATAGTCATTGTAGGATGCCTTGCGCTCCACTGTAGCGATGATGATATGCCAGATTGTCACGCTTAATACGACTCACTAAAGGAGACACTATATGATTCGACTTCATTACAACAAAAGCGTTAAGAATTTCACGGTTCGCCGTGCTGACCGTTCAATCGTATGTGCGAGCGAGCTCCGAGCTAAGATACCTCTTATTGGt                                                                      <  1:7222/301‑1 (MQ=255)`  
                                                                                                                                                                                                                                          `tGTTACTTAGCGGCAGTGTGTGCTTGCGTGTGGAGTATAGTTAACTGGTAATACGACT‑‑CACTAAAGGAGGTACACACCATGATGTACTTAATGCCATTACTCATCGTCATTGTAGGATGCCTTGCGCTCCACTGTAGCGATGATGATATGCCAGATGGTCACGCTTAATACGACTCACTAAAGGAGACACTATATGTTTCGACTTCATTACAACAAAAGCGTTAAGAATTTCACGGTTCGCCGTGCTGACCGTTCAATCGTATGTGCGAGCGAGCGCCGAGCTAAGATACCTCTTATTGGt                                                                      <  1:705598/301‑1 (MQ=255)`  
                                                                                                                                                                                                                                             `tACTTAGCGGCAGTGTGTGCTTGCGTGTGGAGTATAGTTAACTGGTAATACGACT‑‑CACTAAAGGAGGTACACACCATGATGTACTTAATGCCATTACTCATCGTCATTGTAGGATGCCTTGCGCTCCACTGTAGCGATGATGATATGCCAGATGGTCACGCTTAATACGACTCACTAAAGGAGACACTATATGTTTCGACTTCATTACAACAAAAGCGTTAAGAATTTCACGGTTCGCCGTGCTGACCGTTCAATCGTATGTGCGAGCGAGCGCCGAGCTAAGATACATCTTATTGGTAac                                                                   <  1:766982/301‑1 (MQ=255)`  
                                                                                                                                                                                                                                             `ggctcggagatgtgtataagagacaggtgGAGTATAGTTAACTGGTAATACGACT‑‑CACTAAAGGATGTACACACCATGATGTACTTAATGCCATTACTCATCGTCATTGTAGGATGCCTTGCGCTCCACTGTAGCGATGATGATATGCCAGATGGTCATGCTTAATACGACTCCCTAAAGGAGACACTATATGTTTCGACTTCATTACAACAAAAGCGTTAAGAATTTCACGGTTCGCCGTGCTGACCGTTCAATCGTATGTGCGAGCGAGCGCCGAGCTAAGATACCTCTTATTGGTAac                                                                   <  1:164734/275‑1 (MQ=255)`  
                                                                                                                                                                                                                                              `aCTTAGCGGCAGTGTGTGCTTGCGTGCGGAGTATAGTTAACTGGTAATACGACT‑‑CACTAAAGGAGGTACACACCATGATGTACTTAATGCCATTACTCATCGTCATTGTAGGATGCCTTGCGCTCCACTGTAGCGATGATGATATGCCAGATGGTCACGCTTAATACGACTCACTAAAGGAGACACTATATGTTTCGACTTCATTACAACAAAAGCGTTAAGAATTTCACGGTTCGCCGTGCTGACCGTTCAATCGTATGTGCGAGCGAGCGCCGAGCTAAGATACCTCTTAttctgtctc                                                                  >  1:509158/1‑294 (MQ=255)`  
                                                                                                                                                                                                                                               `cTTAGCGGCAGTGTGTGCTTGCGTGTGGAGTATAGTTAACTGGTAATACGACT‑‑CACTAAAGGAGGTACACACCATGATGTACTTAATGCCATTACTCATCGTCATTGTAGGATGCCTTGCGCTCCACTGTATCGATGATGATATGCCAGATGGTCACGCTTAATACGACTCACTAAAGGAGACACTATATGTTTCGACTTCATTACAACAAAAGCGTTAAGAATTTCACGGTTCGCCGTGCTGACCGTTCAATCGTATGTGCGAGCGAGCGCCGAGCTAAGATACCTCTTATTGGAAacac                                                                 >  1:102829/1‑301 (MQ=255)`  
                                                                                                                                                                                                                                               `cTTAGCGGCAGTGTGTGCTTGCGTGTGGAGTATAGTTAACTGGTAATACGACT‑‑CACTAAAGGAGGTACACACCATGATGTACTTAATGCCATTACTAATCGTCATTGTAGGATGCCTTGCGCTCCACTGTAGCGATGATGATATGCCAGATGGTCACGCTTAATACGACTCACTAAAGGAGACACTATATGTTTCGACTTCATTACAACAAAAGCGTTAAGAATTTCACGGTTCGCCGTGCTGACCGTTCAATCGTATGTGCGAGCGAGCGCCGAGCTAAGATACCTATTATTGGTAacac                                                                 <  1:475985/301‑1 (MQ=255)`  
                                                                                                                                                                                                                                                 `tAGCGGCAGTGTGTGCTTGCGTGTGGAGTATAGTTAACTGGTAATACGACT‑‑CACTAAAGGAGGTACACACCATGATGTACTTAATGCCATTACTCATCGTCATTGTAGGATGCCTTGCGCTCCACTGTATCGATGATGATATGCCAGATGGTCACGCTTAATACGACTCACTAAAGGAGACACTATATGTTTCGACTTCATTACAACAAAAGCGTTAAGAATTTCACGGTTCGCCGTTCTGACCGTTCAATCGTATGTGCGAGCGAGCGCCGAGCTAAGATACCTCTTATTGGTAAcacat                                                               >  1:595941/1‑300 (MQ=255)`  
                                                                                                                                                                                                                                                    `gggCAGTGTGTGCTTGCGTGTGGAGTATAGTTAACTGGTAATACGAAT‑‑CACTAAAGGAGGTACACACCATGATGTACTTAATGCCATTACTCATCGTCATTGTAGGATGCCTTGCGCTCCACTGTAGCGATGATGATATGCCAGATGGTCACGCTTAATACGACTCACTAAAGGAGACACTATATGTTTCGACTTCATTACAACAAAAGCGTTAAGAATTTCACGGTTCGCCGTGCTGACCTTTCAATCGTATGTGCGAGCGAGCGCCGAGCTAAGATACCTCTTATTGGTAACACAGTTc                                                            <  1:486899/300‑1 (MQ=255)`  
                                                                                                                                                                                                                                                     `ggCAGTGTGTGCTTGCGTGTGGAGTATAGTTAACTGGTAATACGACT‑‑CACTAAAGGAGGTACACACCATGATGTACTTAATGCCATTACTCATCGTCATTGTAGGATGCCTTGCGCTCCACTGTAGCGATGATGATATGCCAGATGGTCACGCTTAATACGACTCACTAAAGGAGACACTATATGTTTCGACTTCATTACAACAAAAGCGTTAAGAATTTCACGGTTCGCCGTGCTGACCGTTCAATCGTATGTGCGAGCGAGCGCCGAGCTAAGATACCTCTTATTGGTAACACAGTTcc                                                           <  1:545063/301‑1 (MQ=255)`  
                                                                                                                                                                                                                                                           `gtgtgCTTGCGTGTGTAGTATAGTTAACTGGTAATACGACT‑‑CACTAAAGGAGGTACACACCATGATGTACTTAATGCCATTACTCATCGTCATTGTATGATGCCTTGCGCTCCACTGTAGCGATGATGATATGCCAGCTGGTCACGCTTAATACGACTCACTAAATGAGACACTATATGTTTCGACTTCATTACAACAAAAGCGTTAAGAATTTCACGGTTCGCCGTGCTGACCGTTCAATCGTATGTGCGAGCTAGCGCCGAGCTAAGATACCTCTTATTGGtctgtctcttatacacat                                                     >  1:326270/1‑284 (MQ=255)`  
                                                                                                                                                                                                                                                             `gtgCTTGCGTGTGGAGTATAGTTAACTGGTAATACGACT‑‑CACTAAAGGAGGTACACACCATGATGTACTTAATGCCATTACTCATCGTCATTGTAGGATTCCTTGCGCTCCACTGTAGCGATGATGATATGCCAGATGGTCACGCTTAATACGACTCACTAAAGGAGACACTATATGTTTCGACTTCATTACAACAAAAGCGTTAAGAATTTCACGGTTCGCCGTGCTGACCGTTCAATCGTATGTGCGAGCGAGCGCCGAGCTAAGATACCTCTTATTGGTAACACAGTTCCTTTGGCAc                                                   <  1:299483/301‑1 (MQ=255)`  
                                                                                                                                                                                                                                                             `gtgCTTGCGTGTGGAGTATAGTTAACTGGTAATACGACT‑‑CACTAAAGGAGGTACACACCATGATGTACTTAATGCCATTACTCATCGTCATTGTAGGATGCCTTGCGCTCCACTGTAGCGATGATGATATGCCAGATGGTCACGCTTAATACGACTCACTAAAGGAGACACTATATGTTTCGACTTCATTACAACAAAAGCGTTAAGAATTTCACGGTTCGCCGTGCTGACCGTTCAATCGTATGTGCGAGCGAGCGCCGAGCTAAGATAACTCTTATTTGtctgtctcttatacacatct                                                   >  1:558452/1‑282 (MQ=255)`  
                                                                                                                                                                                                                                                                    `cGTGTGGAGTATAGTTAACTGGTAATACGACTCACACTAAAGGCGGTACACACCATGATGTACTTAATGCCATTACTCATCGTCATTGTAGGATGCCTTGCGCTCCACTGTAGCGATGATGATATGCCAGATGGTCACGCTTAATACGACTCACTAAAGGAGACACTATATGTTTCGACTTCATTACAACAAAATCGTTCATAATTTCACGGTTCGCCGTGCTGACCGTTCAATCGTATGTGTGCGCGACCGCCGACCTAATATACCTCTTCTTTTTAACACAGTTCCTTTgtctgtctct                                              >  1:756312/1‑292 (MQ=255)`  
                                                                                                                                                                                                                                                                          `gAGTATAGTTAACTGGTAATACGACT‑‑CACTAAAGGAGGTACACACCATGATGTACTTAATGCCATTACTCATCGTCATTGTAGGATGCCTTGCGCTCCACTGTAGCGATGATGATATGCCAGATGGTCACGCTTAATACGACTCACTAAAGGAGACACTATATGTTTCGACTTCATTACAACAAAAGCGTTAAGAATTTCACGGTTCGCCGTGCTGACCGTTCAATCGTATGTGCGAGCGAGCGCCGAGCTAAGATACCTCTTATTGGTAacacctgtctcttatacacatctccgagccc                                      >  1:306307/1‑274 (MQ=255)`  
                                                                                                                                                                                                                                                                          `gAGTATAGTTAACTGGTAATACGACT‑‑CACTAAAGGAGGTACACACCATGATGTACTTAATGCCATTACTCATCGTCATTGTAGGATGCCTTGCGCTCCACTGTAGCGATGATGATATGCCAGATGGTCACGCTTAATACGACTCACTAAAGGAGACACTATATGTTTCGACTTCATTACAACAAAAGCGTTAAGAATTTCACGGTTCGCCGTGCTGACCGTTCAATCGTATGTGCGAGCGAGCGCCGAGCTAAGATACCTCTTATTGGTAACACAGTTCCTTTGGCACCGAGCGTCcacac                                      >  1:5803/1‑300 (MQ=255)`  
                                                                                                                                                                                                                                                                            `gTATAGTTAACTGGTAATACGACT‑‑CACTAAAGGAGGTACACACCATGATGTACTTAATGCCATTACTCATCGTCATTGTAGGATGCCTTGCGCTCCACTGTAGCGATGATGATATGCCAGATGGTCACGATTAATACGACTCACTAAAGGAGACACTATATGTTTCGACTTCATTACAACAAAAGCGTTAAGAATTTCACGGTTCGCCGTGCTGACCGTTCAATCGTATGTGCGAGCGAGCGCCGAGCTAAGATACCTCTTATTGGTAACACAGTTCCTTTGGCACCGAGCGTCCAcatca                                    >  1:141289/1‑301 (MQ=255)`  
                                                                                                                                                                                                                                                                              `ataGTTAACTGGTAATACGACT‑‑CACTAAAGGAGGTACACACCATGATGTACTTAATGCCATTACTCATCGTCATTGTAGGATGCCTTGCGCCCCACTGTAGCGATGATGATATGCCAGATGGTCACGCTTAATACGACTCACTAAAGGAGACACTATATGTTTCGACTTCATTACAACAAAAGCGTTAAGAATTTCACGGTTCGCCGTGCTGACCGTTCAATCGTATGTGCGAGCGAGCGCCTAGCTAAGATACCTCTTATTGGTAACACCGTTCCTTTGGCACCGAGCGTCCACATCAtt                                  >  1:420356/1‑301 (MQ=255)`  
                                                                                                                                                                                                                                                                                `aGTTAACTGGTAATACGACT‑‑AACTAAAGTAGGTACACACCATGATGTACTTAATGCCATTACTCATCGTCATTGTAGGATGCCTTGCGCTCCACTGTAGCGATGATGATATGCCAGATGGTAACGCTTAATACGACTCACTAAAGGAGACACTATATGTTTCGACTTCATTACAACAAAAGCGTTAAGAATTTCACGGTTCGCCGTGCTGACCGTTCAATCGTATGTGCGAGCGAGCGCCGAGCTAAGATACCTCTTATTGGTAACACAGTTCCTTTGGCACCGAGCGTCCACATCATTAt                                <  1:297375/301‑1 (MQ=255)`  
                                                                                                                                                                                                                                                                                 `gTTAACTGGTAATACGACT‑‑CACTAAAGGAGGTACACACCATGATGTACTTAATGCCATTACTCATCGTCATTGTAGGATGCCTTGCGCTCCACTGTAGCGATGATGATATGCCAGATGGTCACGCTTAATACGACTCACTAAAGGAGACACTATATGTTTCGACTTCATTACAACAAAAGCGTTAAGAATTTCACGGTTCGCCGTGCTGACCATTCAATCGTATGTGCGAGCGAGCGCCGAGCTAAGATACCTCTTATTGGTAACACAGTTccctgtctcttatacacatctccgagccca                               >  1:412526/1‑273 (MQ=255)`  
                                                                                                                                                                                                                                                                                 `gTTAACTGGTAATACGACT‑‑CACTAAAGGAGGTACACACCATGATGTACTTAATGCCATTACTCATCGTCATTGTAGGATGCCTTGCGCTCCACTGTAGCGATGATGATATGCCAGATGGTCACGCTTAATACGAATCACTAAAGGAGACACTATATGTTTCGACTTCATTACAACAAAAGCGTTAAGAATTTCACGGTTCGCCGTGCTGACCGTTCAATCGTATGTGCGAGCGAGCGCCGAGCTAAGATACCTCTTATTGGTAACACAGTTCCTTTGGCACCGAGCGTCCACATCATTAtt                               >  1:201935/1‑300 (MQ=255)`  
                                                                                                                                                                                                                                                                                    `aaCTGGTAATACGACT‑‑CACTAAAGGAGGTACACACCATGATGTACTTAATGCCATTACTCATCGTCATTGTAGGATGCCTTGCGCTCCACTGTAGCGATGATGATATGCCAGATGGTCACGCTTAATACGACTCACTAAAGGAGACACTATATGTTTCGACTTTATTACAACAAAAGCGTTAAGAATTTCAAGGTTCGCCGTGCTGACAGTTCAATCGTATGTGCAAGCGAGCGCCGAGCTAAGATACCTCTTATTGGTAACACAGTTCCTTTGGCACCGAGCGTCCACATCATTATCAcc                            <  1:338580/301‑1 (MQ=255)`  
                                                                                                                                                                                                                                                                                    `aaCTGGTAATACGACT‑‑CACTAAAGGAGGTACACACCATGATGTACTTAATGCCATTACTCATCGTCATTGTAGGATGCCTTGCGCTCCACTGTAGCGATGATGATATGCCAGATGGTCACGCTTAATACGACTCACTAAAGGAGACACTATATGTTTCGACTTCATTACAACAAAAGCGTTAAGAATTTCACGGTTCGCCGTGCTGACCGTTCAATCGTATGTGCGAGCGAGCGCCGAGCTAAGATACCTCTTATTGGTAACACAGTTCCTTTGGCACCGAGCGTCCACATCATTATCAcc                            <  1:122058/301‑1 (MQ=255)`  
                                                                                                                                                                                                                                                                                      `cTGGTAATACGACT‑‑CACTAAAGGAGGTACACACCATGATGTACTTAATGCCATTACTCATCGTCATTGTAGGATGCCTTGCGCTCCACTGTAGCGTTGATGATATGCCAGATGGTCACGCTTAATACGACTCACTAAAGGAGACACTATATGTTTCGACTTCATTACAACAAAAGCGTTAAGAATTTCACGGTTCGCCGTGCTGGCCGTTCAATCGTATGTGCGAGCGAGCGCCGAGCTAAGATACCTCTTATTGGTAACACAGTTCCTTTGGcctgtctcttatacacatctccgagccc                          >  1:315329/1‑274 (MQ=255)`  
                                                                                                                                                                                                                                                                                       `atgtgtataagagacagcTAAAGGAGGTACACACCATGATGTACTTAATGCCATTACTCATCGTCATTGTAGGATGCCTTGCGCTCCACTGTAGCGATGATGATATGCCAGATGGTCACGCTTAATACGACTCACTAAATGAGACACTATATGTTTCGACTTCATTACAACAAAAGCGTTAAGAATTTCACGGTTCGCCGTGCTGACCGTTCAATCGTATGTGCGAGCGAGCGCCGAGCTAAGATACCTCTTATTGGTAACACAGTTCCTTTGGCACCGAGCGTCCACATCATTATCAccc                           <  1:156616/284‑1 (MQ=255)`  
                                                                                                                                                                                                                                                                                          `tAATACGACT‑‑CACTAAAGGAGGTACACACCATGATGTACTTAATGCCATTACTCATCGTCATTGTAGGATGCCTTTCGCTCCACTGTAGCGATGATGATATGCCAGATGGTCACGCTTAATACGACTCACTTAAGGAGACACTATATGTTTCGACTTCATTACAACAAAAGCTTTAAGAATTTCACGTTTCGCCGTGCTGACCGTTCAATCGTATGTGCGAGCGAGCGCCTTGCTAAGCTACCTCTTATTGGTAACACAGTTCCTTTGGCCACGAGCGTCCACATCATTTACACCCgttgt                      >  1:695212/1‑301 (MQ=255)`  
                                                                                                                                                                                                                                                                                           `aaTACGACT‑‑CACTAAAGGAGGTACACACCATGATGTACTTAATGCCATTACTCATCGTCATTGTAGGATGCCTTGCGCTCCACTGTAGCGATGATGATATGCCAGATGGTCACGCTTAATACGACTCACTAAAGGAGACACTATATGTTTCGACTTCATTACAACAAAAGCGTTAAGAATTTCACGGTTCGCCGTGCTGACCGTTCAATCGTATGTGCGAGCGAGCGCCGAGCTAAGATACCTCTTATTGGTAACACAGTTCCTTTGGCACCGAGCGTCCACATCATTATCACCCgtggtg                     <  1:478217/301‑1 (MQ=255)`  
                                                                                                                                                                                                                                                                                                      `cACTAAATGAGGTACACACCATGATGTACTTAATGCCATTACTCATCGTCATTGTAGGATGCCTTGCGCTCCACTGTAGCGATGATGATATGCCAGATGGTCACGCTTAATACGACTAACTAAAGGAGACACTATATGTTTCGACTTCATTACAACAAAAGCGTTAAGAATTTCACGGTTCGCCGTGCTGACCGTTCAATCGTATGTGCGAGCGAGCGCCGAGCTACGATACCTCTTATTGGTAACACAGTTCCTTTGGCACCGAGCGTCCACATCATTATCACCCGTGGTGACTTTgaga            >  1:183890/1‑301 (MQ=255)`  
                                                                                                                                                                                                                                                                                                      `cACTAAAGGAGGTACACACCATGATGTACTTAATGCCATTACTCATCGTCATTGTAGGATGCCTTGCGCTCCACTGTAGCGATGATGATATGCCAGATGGTCACGCTTAATACGACTCACTAAAGGAGACACTATATGTTTCGACTTCATTACTACAAAAGCGTTAAGAATTTCACGTTTCGCCGTGCTGACCGTTCAATCGTATGTGCGAGCGAGCGCCGAGCTAAGATACCTCTTATTGGTAACACAGTTCCTTTGTCACCGAGCGTCCACATCATTATCAcccctttctcttatacaa            >  1:349850/1‑286 (MQ=255)`  
                                                                                                                                                                                                                                                                                                      `cACTAAAGGAGGTACACACCATGATGTACTTAATGCCATTACTCATCGTCATTGTAGGATGCCTTGCGCTCCACTGTAGCGATGATGATATGCCAGATGGTCACGCTTAATACGACTCACTAAAGGAGACACTATATGTTTCGACTTCATTACAACAAAAGCGTTAAGAATTTCACGGTTCGCCGTGCTGACCGTTCAATCGTATGTGCGAGCGAGCGCCGAGCTAAGATACCTCTTATTGGTAACACAGTTCCTTTGGCACCGAGCGTCCACATCATTATCACCCGTGGTGACTTTgagc            >  1:128498/1‑300 (MQ=255)`  
                                                                                                                                                                                                                                                                                                      `cACTAAAGGAGGTACACACCATGATGTACTTAATGCCATTACTCATCGTCATTGTAGGATGCCTTGCGCTCCACTGTAGCGATGATGATATGCCAGATGGTCACGCTTAATACGACTCACTAAAGGAGACACTATATGTTTCGACTTCATTACAACAAAAGCGTTAAGAATTTCACGGTTCGCCGTGCTGACCGTTCAATCGTATGTGCGAGCGAGCGCCGAGCTAAGATACCTCTTATTGGTAACACAGTTCCTTTGGCACCGAGCGTCCACATCATTATCACCCGTGGTGACTTTgaga            >  1:17996/1‑301 (MQ=255)`  
                                                                                                                                                                                                                                                                                                        `cTAAAGGAGGTACACACCATGATGTACTTAATGCCATTACTCATCGTCATTGTAGGATGCCTTGCGCTCCACTGTAGCGATGATGATATGCCAGATGGTCACGCTTAATATGACTCACTAAAGGAGACACTATATGTTTCGACTTCATTACAACAAAAGCGTTAAGAATTTCACGGTTCGCCGTCCTGACCGTTCAATCGTATGTGCGAGCTAGCGCCGAGTTAAGATACCTCTTATTGGTAACACAGTTCCTTTGGCACCGAGCGTCTACATCATTATctgtctcttatacacatttccc          >  1:627322/1‑280 (MQ=255)`  
                                                                                                                                                                                                                                                                                                               `aggTACACACCATGATGTACTTAATGCCATTACTCATCGTCATTGTAGGATGCCTTGCGCTCCACTGTAGCGATGATGATATGCCAGATGGTCACGCTTAATACGACTCACTAAAGGAGACACTATATGTTTCGACTTCATTACAACAAAAGCGTTAAGAATTTCACGGTTCGCCATGCTGACCGTTCAATCGTATGTGCGAGCGAGCGCCGAGCTAAGATACCTCTTATTGGTAACACAGTTCCTTTGGCACCGAGCGTCCACATCATTATCACCCGTGGTGACTTTGAGAAAGCAATAg   <  1:517764/301‑1 (MQ=255)`  
                                                                                                                                                                                                                                                                                                                `ggTACACACCATGATGTACTTAATGCCATTACTCATCGTCATTGTAGGATGCCTTGCGCTCCACTGTAGCGATGATGATATTCCAGATGGTCACGCTAAATACGACTCACTAAAGGAGACACTATATGTTTCGACTTCATTACAACAAAAGCGTTAAGAATTTCACGGTTCGCCGTGCTGACCGTTCAATCGTATGTGCGAGCGAGCGCCGAGCTAAGATACCTCTTATTGGTAACACAGTTCCTTTGGCACCGAGCGTCCACATCATTATCACCCGTGGTGACTTTGAGAAAGCAATAGa  >  1:539419/1‑301 (MQ=255)`  
                                                                                                                                                                                                                                                                                                                `ggTACACACCATGATGTACTTAATGCCATTACTCATCGTCATTGTAGGATGCCTTGCGCTCCACTGTAGCGATGATGATATGCCAGATGGTCACGCTTAATACGACTCACTAAAGGATACACTATATGTTTCGACTTCATTACAACAAAAGCGTTAAGAATTTCACGGTTCGCCGTGCTGACCGTTCAATCGTATGTGCGAGCGAGCGCCGAGCTAAGATACCTCTTATTGGTAACACAGTTCCTTTGGCACCGAGCGTCCACATCATTATCACCCGTGGTGACTTTGAGAAAGCAATAGa  >  1:398543/1‑301 (MQ=255)`  

                                                                                                                                                                                                                                                                                                                `|`                                                                                                                                                                                                                                                                                                              
`CTGATGGCTCTTTGCGGCACCCATCGTTCGTAATGTTCCGTGGCACCGAGGACAACCCTCAAGAGAAAATGTAAT‑‑CACACTGGCTCACCTTCGGGTGGGCCTTTCTGCGTTTATAAGGAGACACTTTATGTTTAAGAAGGTTGGTAAATTCCTTGCGGCTTTGGCAGCTATCCTGACGCTTGCGTATATTCTTGCGGTATACCCTCAAGTAGCACTAGTAGTAGTTGGCGCTTGTTACTTAGCGGCAGTGTGTGCTTGCGTGTGGAGTATAGTTAACTGGTAATACGACT‑‑CACTAAAGGAGGTACACACCATGATGTACTTAATGCCATTACTCATCGTCATTGTAGGATGCCTTGCGCTCCACTGTAGCGATGATGATATGCCAGATGGTCACGCTTAATACGACTCACTAAAGGAGACACTATATGTTTCGACTTCATTACAACAAAAGCGTTAAGAATTTCACGGTTCGCCGTGCTGACCGTTCAATCGTATGTGCGAGCGAGCGCCGAGCTAAGATACCTCTTATTGGTAACACAGTTCCTTTGGCACCGAGCGTCCACATCATTATCACCCGTGGTGACTTTGAGAAAGCAATAGA  >  NC_001604.1/7481‑8081`

  

Alignment Legend

Aligned base mismatch/match (shaded by quality score): `ATCG/ATCG` `< 3 ≤` `ATCG/ATCG` `< 15 ≤` `ATCG/ATCG` `< 28 ≤` `ATCG/ATCG` `< 37 ≤` `ATCG/ATCG` `< 38 ≤` `ATCG/ATCG`

Unaligned base: `atcg`    Masked matching base: `atcg`    Alignment gap: `‑`    Deleted base: `‑`

Reads not counted as support for junction

read\_name Not counted due to insufficient overlap past the breakpoint.

read\_name Not counted due to not crossing MOB target site duplication.
